# *Lactobacillus bulgaricus*, *Lactobacillus rhamnosus* and *Lactobacillus paracasei* Attenuate *Salmonella* Enteritidis, *Salmonella* Heidelberg and *Salmonella* Typhimurium Colonization and Virulence Gene Expression In Vitro

**DOI:** 10.3390/ijms18112381

**Published:** 2017-11-09

**Authors:** Muhammed Shafeekh Muyyarikkandy, Mary Anne Amalaradjou

**Affiliations:** Department of Animal Science, University of Connecticut, Storrs, CT 06269, USA; muhammed.muyyarikkandy@uconn.edu

**Keywords:** *Salmonella*, lactic acid bacteria, probiotic, cecal colonization, macrophages, gene expression, in vitro

## Abstract

*Salmonella* Enteritidis (SE), *Salmonella* Typhimurium (ST), and *Salmonella* Heidelberg (SH) have been responsible for numerous outbreaks associated with the consumption of poultry meat and eggs. *Salmonella* colonization in chicken is characterized by initial attachment to the cecal epithelial cells (CEC) followed by dissemination to the liver, spleen, and oviduct. Since cecal colonization is critical to *Salmonella* transmission along the food chain continuum, reducing this intestinal association could potentially decrease poultry meat and egg contamination. Hence, this study investigated the efficacy of *Lactobacillus delbreuckii* sub species *bulgaricus* (NRRL B548; LD), *Lactobacillus paracasei* (DUP-13076; LP), and *Lactobacillus rhamnosus* (NRRL B442; LR) in reducing SE, ST, and SH colonization in CEC and survival in chicken macrophages. Additionally, their effect on expression of *Salmonella* virulence genes essential for cecal colonization and survival in macrophages was evaluated. All three probiotics significantly reduced *Salmonella* adhesion and invasion in CEC and survival in chicken macrophages (*p* < 0.05). Further, the probiotic treatment led to a significant reduction in *Salmonella* virulence gene expression (*p* < 0.05). Results of the study indicate that LD, LP, and LR could potentially be used to control SE, ST, and SH colonization in chicken. However, these observations warrant further in vivo validation.

## 1. Introduction

*Salmonella enterica*, a Gram-negative foodborne pathogen, is one of the leading causes of foodborne gastroenteritis in humans. Globally, it is estimated that foodborne Salmonellosis accounts for 93.8 million cases and 155,000 deaths each year [1]. In the United States, the Centers for Disease Control and Prevention (CDC) estimates that *Salmonella* is responsible for 1 million illnesses, and 380 deaths annually [2]. Although different *Salmonella* serovars have been implicated in these foodborne outbreaks, a limited number of them are responsible for most human infections [3,4]. In the US, during 2007–2011, five of the most common serovars caused 61% of all *Salmonella*-related outbreaks. Serovar Enteritidis was the most frequently isolated (27%) followed by Typhimurium (14%), Newport (10%), Heidelberg (7%), and Montevideo (3%) [5].

While various food sources, including pork, beef, and fresh produce, have been implicated in *Salmonella* outbreaks, these infections are often associated with the consumption of raw or undercooked poultry products [3,6]. In fact more than 70% of human Salmonellosis in the US are attributed to the consumption of contaminated poultry meat and eggs [5]. Further investigations into the serovar diversity within each commodity group revealed that the egg- and chicken-associated outbreaks were predominantly caused by *S*. Enteritidis (SE, 83%) and *S*. Heidelberg (SH; 9%), and SE (28%), *S*. Typhimurium (ST; 23%), and SH (13%), respectively [4]. This link between zoonotic foodborne infection and poultry products is concerning given the tremendous increase in the demand for poultry meat and eggs. It is estimated that people consume approximately 250 eggs per year and that poultry meat constitutes almost 50% of the annual per capita consumption of meat in the US [6,7]. Therefore, the microbiological safety of poultry products is a major concern from public health and economic perspectives [8].

Poultry populations, particularly chickens are frequently colonized with *Salmonella* by horizontal and vertical transmission [3,9,10]. On the farm, the contamination cycle begins with the infection of the animal and proceeds by shedding of pathogens in the feces which, in turn, contaminates the environment and leads to new infections or reinfection of animals [3,6]. In chickens, within a few hours of oral infection, *Salmonella* can colonize and invade the ceca, reaching other internal organs like the liver and spleen [10,11]. In laying hens, systemic dissemination includes the colonization and invasion of the reproductive tissues thereby resulting in direct contamination of eggs [10,12]. Therefore, intestinal colonization is central to *Salmonella* entry into the food chain continuum either via contaminated meat and/or eggs [13]. Consequently, pre-harvest control measures aiming at reducing *Salmonella* colonization are critical to prevent pathogen contamination on poultry products and promote public health [14,15,16].

Various strategies including sanitary barriers, acidification of the chicken environment using short- and medium-chain fatty acids, vaccination, bacteriophages, antimicrobial peptides, and probiotics, have been investigated for their ability to reduce *Salmonella* in chicken [17]. Among the antimicrobial approaches mentioned above, probiotics including lactic acid bacteria (LAB) have shown promise in the control of poultry pathogens [18]. Probiotics are defined as “live microorganisms that, when administered in adequate amounts, confer a health benefit on the host” [19]. Probiotics reduce survival and colonization of enteric pathogens through several mechanisms including improved intestinal barrier function, competitive exclusion, production of antimicrobial metabolites, and the modulation of the structure and function of intestinal epithelium [20]. In this regard, the use of LAB has been suggested as an effective measure to control salmonellosis in chicken [21]. Additionally, in-feed supplementation of probiotic LAB have demonstrated growth-promoting abilities associated with an increase in growth and performance in chicken [22]. Moreover, these studies have shown that supplementation of probiotics to chicken may reduce dysbiosis and help develop natural resistance to pathogens [23,24]. In light of the need for effective alternatives to control *Salmonella* in chicken, our study investigated the probiotic potential of select LAB to inhibit the attachment to and invasion of chicken primary cecal epithelial cells (CEC) by SE, ST, and SH in vitro. Additionally, the effect of LAB on the expression of virulence genes essential for *Salmonella* colonization in the cecum was studied.

## 2. Results and Discussion

While there are several opportunities for transfer of *Salmonella* to poultry meat and eggs, cecal colonization in chickens is central to the direct transmission of the pathogen along the food chain continuum [25,26,27]. Therefore, reducing cecal carriage in chicken would help control fecal shedding and eventual contamination of poultry carcass and eggs. Towards this, our research focusses on the development of probiotic-based prophylactics to control *Salmonella* in chicken. As a first step, in the current study, we determined the efficacy of *L. rhamnosus* NRRL B442 (LR), *L. paracasei* DUP-13076 (LP) and *L. delbrueckii* sub sp. *bulgaricus* NRRL B548 (LD) to inhibit *Salmonella* colonization and dissemination in vitro. To date, no studies have investigated the probiotic potential of LR, LP, and LD in controlling *Salmonella* colonization.

### 2.1. Sub-Inhibitory Concentrations (SICs) of LAB Cell-Free Supernatant (CFSN)

The highest concentration of CFSN which did not inhibit *Salmonella* growth was determined to be the SIC. Of the different CFSN concentrations tested, 7.5% CFSN was identified to be the SIC for LP, LD, and LR. The average initial *Salmonella* (SE/ST/SH) population in the control and CFSN-treated samples was ~6.3 log_10_ CFU/mL. Following incubation at 37 °C for 24 h, ~8.2 log_10_ CFU/mL of *Salmonella* spp. was recovered from all samples, irrespective of control and CFSN treatment. This confirmed that the CFSN concentration used in the assay (7.5%) was not inhibitory to the bacteria. Since the LAB tested were found to be equally effective against different isolates within each *Salmonella enterica* serovar, namely SE isolates (SE-21 and SE-90), ST isolates (ST-43 and ST-J380) and SH isolates (SH-1 and SH-V6FA), for any of the tested parameters, only results observed with SE-90, ST-43, and SH-V6FA are provided in the manuscript, unless mentioned otherwise.

### 2.2. Motility Assay

**Figure 1:**
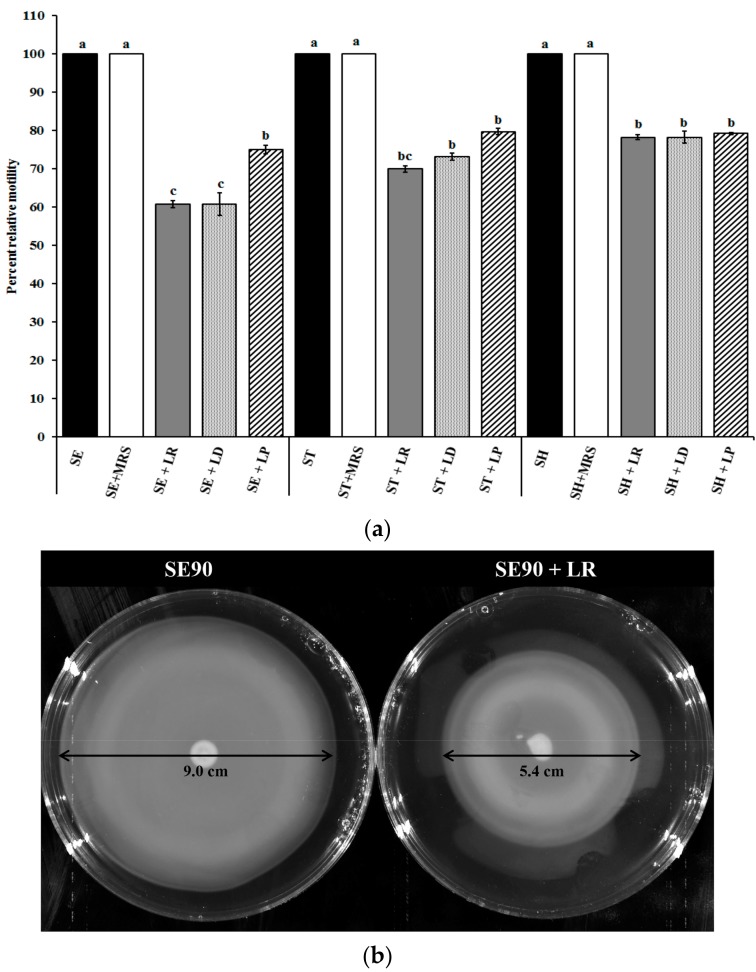

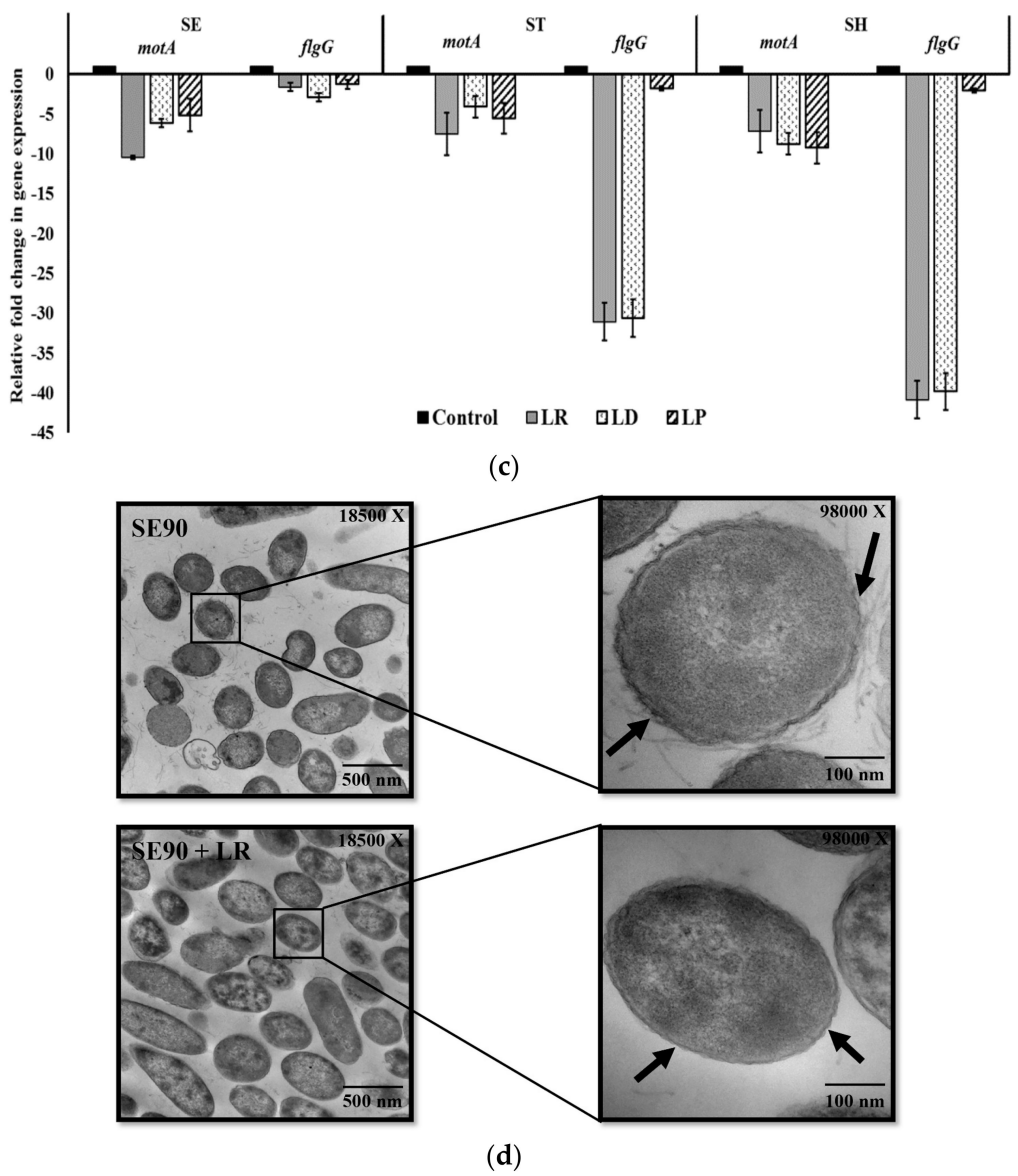
(**a**) Effect of sub-inhibitory concentrations of LAB supernatants on *Salmonella* motility. Data are presented as means ± SEM. ^a−c^ Different superscripts indicate the significant difference in LS-means (*p* < 0.05), MRS-de Man, Rogosa and Sharpe broth, SE: *S*. Enteritidis 90; ST: *S*. Typhimurium DT104 43; SH: *S*. Heidelberg V6FA; LR: *L. rhamnosus* NRRL B442; LP: *L. paracasei* DUP-13076; LD: *L. delbreuckii bulgaricus* NRRL B548; (**b**) Representative image of the motility assay performed with *Salmonella* Enteritidis 90 (SE90) treated with and without SIC of *L. rhamnosus* NRRL B442 (LR) supernatant; (**c**) Effect of sub-inhibitory concentrations of LAB supernatants on the expression of motility genes in *Salmonella*. Data are presented as means ± SEM; (**d**) Representative TEM images of *Salmonella* Enteritidis 90 (SE90) treated with and without SICs of *L. rhamnosus* NRRL B442 (LR) supernatant. *Salmonella*, Arrows indicate the presence and absence of flagella.

Since bacterial motility is a key attribute that enables intestinal colonization by the pathogen [28], we evaluated the ability of different LAB cultures to inhibit *Salmonella* motility. Briefly, SE, ST, and SH were grown in the presence of SICs of LAB CFSN (7.5%), and the motility assay was performed. It can be seen from Figure 1 that LP, LR, and LD significantly (*p* < 0.05) reduced *Salmonella* motility, by 20–40% compared to the control (Figure 1a). Treatment with LR reduced SE, ST, and SH motility by 40%, 30%, and 20%, respectively, compared to the control. Similarly, LD reduced ST motility by ~25%, while LP resulted in only ~15% reduction in ST motility. In the case of SH, LR, LD, and LP were found to be equally effective in reducing pathogen motility by 20%. Figure 1b is a pictorial representation of the observed reduction in SE motility following treatment with LR CFSN.

As can be seen from the Figure 1b, treatment with LR CFSN reduced the zone of motility to 5.4 ± 0.16 cm compared to 9.0 ± 0.08 cm in the untreated control group (~40% reduction). This reduction in motility could be either due to a defect in structure or function of the *Salmonella* motility apparatus [29,30,31]. To understand the underlying molecular mechanism, real-time quantitative PCR (RT-qPCR) was performed to evaluate the differential expression of genes associated with motility in *Salmonella*, namely, *flgG* and *motA*. While MotA is important in providing the motive force to the flagella, FlgG is critical to the formation of the flagellar rod [32,33]. Results of this assay revealed that LR, LD, and LP significantly reduced *motA* and *flgG* expression in SE, ST, and SH compared to the untreated control (*p* < 0.05; Figure 1c). For example, LR reduced *motA* expression by ~7–10 fold in all serovars, and *flgG* expression by 2-, 30-, and 40-fold in SE, ST, and SH, respectively. In order to detect any structural changes in the flagellar apparatus induced by LAB treatment, transmission electron microscopy was performed. As can be seen from Figure 1d, treatment of SE with LR CFSN reduced the flagellar presence on the cell surface when compared to the untreated control. These results are in conjunction with previous studies that have demonstrated that reduced expression of genes associated with motility will impair the formation of the motility apparatus [34].

### 2.3. Inhibition of Salmonella Adhesion and Invasion in Cecal Epithelial Cells (CEC)

**Figure 2:**
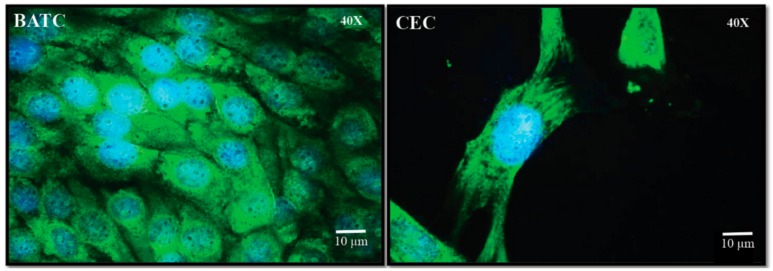
Confirmation of epithelial characteristics of CEC using immunofluorescence.

Since no established chicken cecal epithelial cell lines (CEC) are commercially available, we isolated primary epithelial cells from chicken ceca. The primary CEC have been previously utilized to study *Salmonella* colonization and interaction with the chicken cecal epithelium [35]. Epithelial characterization of the isolated CEC was confirmed by selectively staining for cytokeratin using FITC labeled anti-cytokeratin antibodies (Figure 2) [35]. Furthermore, to validate the epithelial localization of cytokeratin, immunostaining of Budgerigar abdominal tumor cells (BATC, permanent avian intestinal epithelial cell line) was performed as the positive control.

Following validation, the monolayer was utilized in the adhesion and invasion assays. Before proceeding with the assays, LAB candidates (LD, LP, and LR) were screened for their ability to adhere to the CEC. Adhesion to the intestinal epithelium is a critical probiotic attribute that facilitates competitive exclusion and antagonism against pathogens [36]. The select LAB cultures (7 log_10_ CFU/mL) were added to the CEC monolayer and incubated for 2 h. Following incubation, enumeration of the adhered LAB was performed. Approximately 6 log_10_ CFU/mL of adhered LAB was recovered with all the isolates tested. In addition to adhesion, the absence of any adverse effects on the CEC due to LAB pre-exposure was verified using the trypan blue assay. This assay revealed that LD/LP/LR did not exert any adverse effects on CEC viability, and no cytotoxicity was observed (data not shown). Further, there was no significant difference (*p* > 0.05) between the adhesion and invasion capabilities of the different *Salmonella* isolates used in this study (data not shown).

**Figure 3:**
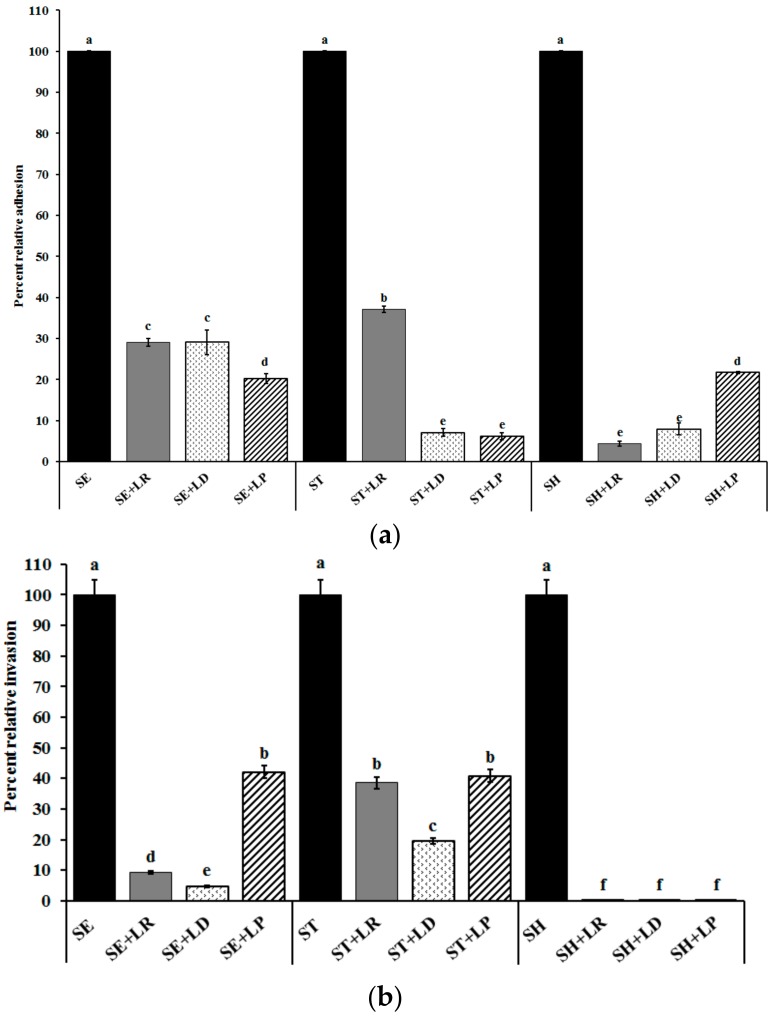
(**a**) Effect of LAB pre-treatment on *Salmonella* adhesion to primary cecal epithelial cells. *Salmonella*; ^a−e^ Different superscripts indicate the significant difference in LS-means (*p* < 0.05); (**b**) Effect of LAB pre-treatment on *Salmonella* invasion in primary cecal epithelial cells. *Salmonella*; Data are represented as means ± SEM; ^a−f^ Different superscripts indicate the significant difference in LS-means (*p* < 0.05).

For the *Salmonella* adhesion inhibition assay, CEC monolayers were pre-exposed to the different LAB cultures (LD/LP/LR) for 24 h followed by *Salmonella* infection. Figure 3a depicts the results of the *Salmonella* adhesion assay. The results of this assay demonstrate that LD, LP, and LR were effective in significantly (*p* < 0.05) inhibiting SE, ST, and SH adhesion to primary CECs by ~60–95% when compared with the control (Figure 3a). In addition to inhibiting adhesion, LR, LD, and LP significantly reduced *Salmonella* invasion into CEC by ~60–99% (Figure 3b). Although all three LAB cultures reduced *Salmonella* adhesion and invasion in CEC, this inhibitory effect was found to be species-specific with respect to the LAB cultures tested. For example, while LR and LD were equally effective in reducing SE invasion by >90%, exposure to LP only resulted in 60% inhibition of pathogen internalization (Figure 3b). Furthermore, the LAB mediated anti-*Salmonella* activity was also found to vary significantly (*p* < 0.05) with the *Salmonella* serovar with differences observed between SE, ST, and SH (Figure 3).

To understand the underlying mechanism that mediates LAB-associated attenuation of *Salmonella* colonization, we performed RT-qPCR on *Salmonella* virulence genes. These include genes for adhesion and invasion, namely, *sopB* and *invH* [37,38]; Type III secretion system (T3SS) effectors, namely, *sipA and sipB* [38]; and *Salmonella* pathogenicity island-I (SPI-I) encoded transcriptional regulators *hilA* and *hilD* [39]. *Salmonella* outer protein B (encoded by *sopB*) is a lipid phosphatase that has been demonstrated to be critical for enteropathogenicity in a bovine model [40,41]. SPI-1 of *Salmonella* encodes two transcriptional regulators, HilA, and InvH; HilA regulates expression of T3SS machinery, and InvH is required for proper assembly of T3SS-1. Moreover, HilD is known to modulate the expression of *hilA* [42,43,44]. Additionally, Shah and others [45] demonstrated that *hilD* and *invH* mutants were associated with attenuated virulence in vivo*.* Additionally, deletion of *invH* in *Salmonella* resulted in a reduced injection of T3SS effector proteins into the host cells [46]. Hence, downregulation of these genes could lead to reduced adhesion and invasion of *Salmonella* into the host cells. Results of our RT-qPCR assays demonstrate that following LAB treatment, all the genes tested showed a significant reduction in their expression (*p* < 0.05) when compared to the control (Table 1). Genes critical to adhesion including *invH*, *hilA*, and *hilD*, were downregulated by ~2–12 fold by LP, LD, and LR in all *Salmonella* serovars. Similarly, a significant reduction in the expression *sipA, sipB*, and *sopB* was also observed with SE, ST, and SH. These results corroborate with the cell association assays where exposure to LAB significantly reduced adhesion and invasion of SE, ST, and SH in CECs (Figure 3).

### 2.4. Inhibition of Salmonella Invasion and Survival in Chicken Macrophage

**Figure 4:**
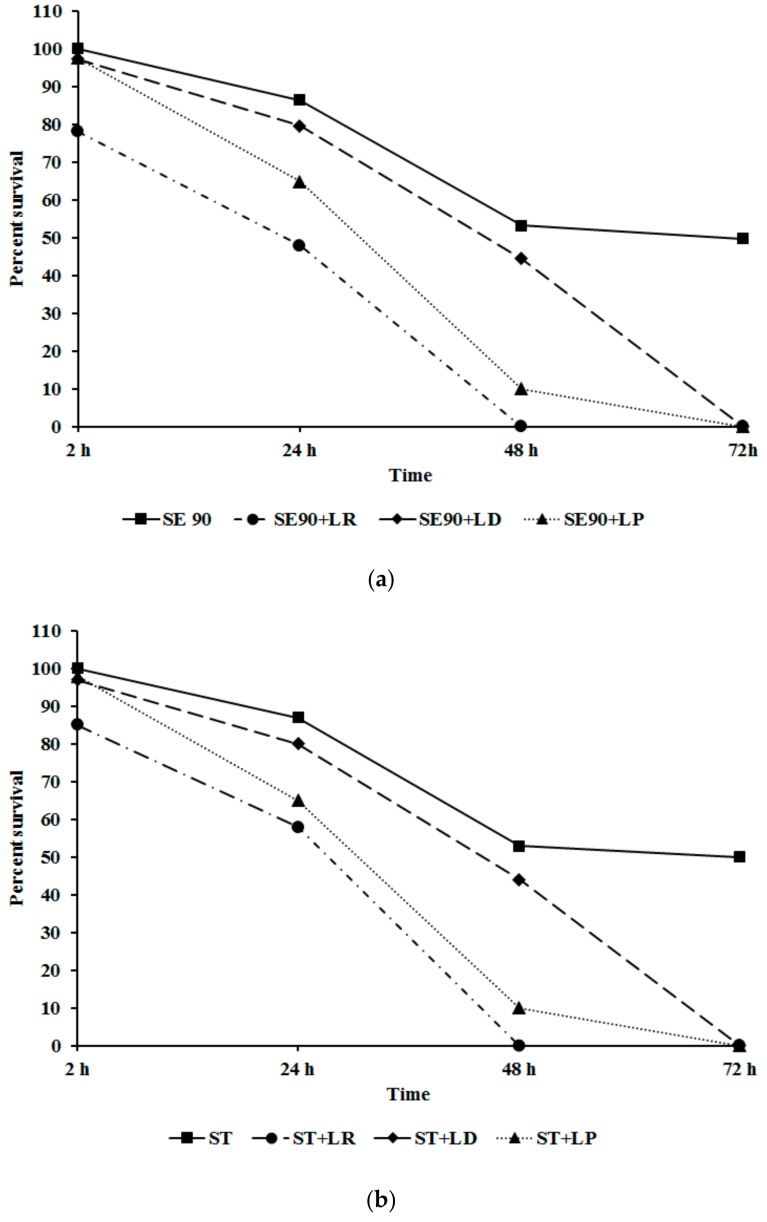

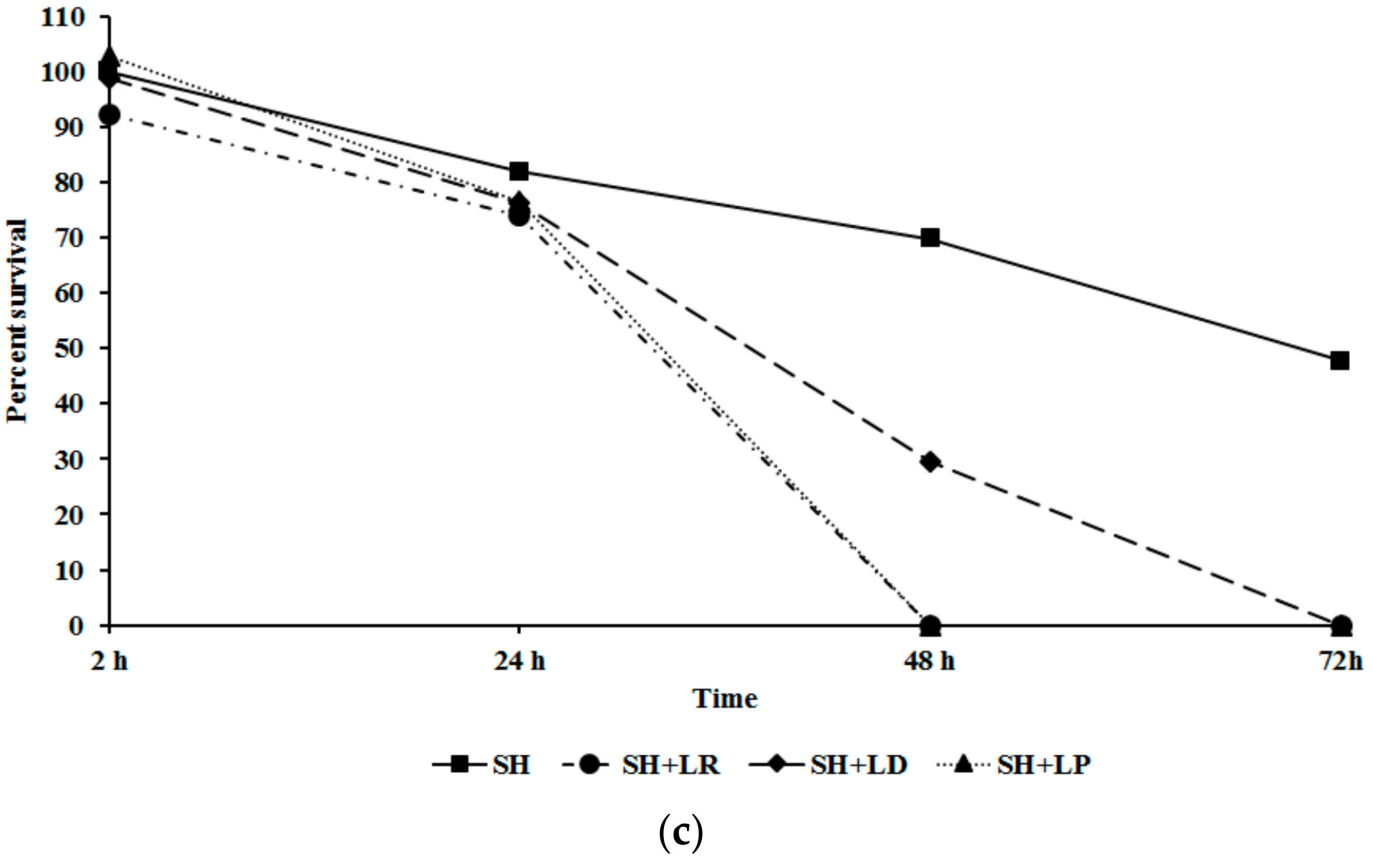
(**a**) Effect of LAB pre-treatment on *Salmonella* Enteritidis 90 invasion and survival in chicken macrophages. Data are represented as means ± SEM; (**b**) Effect of LAB pre-treatment on *Salmonella* Typhimurium DT 104 43 invasion and survival in chicken macrophages. Data are represented as means ± SEM; (**c**) Effect of LAB pre-treatment on *Salmonella* Heidelberg V6FA invasion and survival in chicken macrophages. Data are represented as means ± SEM.

Following intestinal colonization, *Salmonella* is disseminated to other organs including the liver, spleen, and oviduct by macrophages [12,47]. Hence, we investigated the ability of LAB to inhibit *Salmonella* invasion and survival in chicken macrophages. It can be seen from Figure 4 that the different LAB CFSNs at their SICs (7.5%) were effective in significantly reducing *Salmonella* invasion and survival in chicken macrophages (*p* < 0.05). For example, LR significantly reduced *Salmonella* survival by 50%, 29%, and 25% by 24 h with SE, ST, and SH, respectively, with complete inhibition observed at the end of the study (Figure 4) A similar trend was observed with LP and LD in the case of SE and ST, however, with SH, treatment with LR and LP completely inhibited its intracellular survival by 48 h (Figure 4).

As previously discussed, following macrophage invasion, *Salmonella* successfully spreads to extra-intestinal tissues resulting in systemic dissemination. Effector proteins of SPI-2 T3SS, such as SpvB, are critical to the survival of *Salmonella* in macrophages [48]. In addition to SpvB, SPI-1 T3SS effectors, SipA modulates host actin cytoskeleton, and SopB protects the *Salmonella*-infected epithelial cells from phagocytosis [49]. About 2–15 fold reduction in *sipA* and *sopB* expression was noticed in treatment groups (Table 1). SipB of SPI-1 T3SS has been demonstrated to bind to caspase 1 leading to bovine and murine macrophage cell death, thereby promoting *Salmonella* survival and escape from the phagocytic cells [41]. Our study has demonstrated that LP, LR, and LD reduced *SipB* and *SpvB* expression by ~2 to 30-fold thereby preventing *Salmonella* escape and reducing its survival in macrophages. Further, the reduced expression of *sipB* could lead to the observed reduction in the intracellular survival of *Salmonella* in HTC (Figure 4). Results from the current study demonstrate that application of LP, LD, and LR significantly impaired the pathogen’s ability to invade and survive within chicken macrophages by modulating virulence gene expression in the SPI-1 and SPI-II T3SS locus.

## 3. Materials and Methods

### 3.1. Bacterial Isolates and Growth Conditions

LAB used in the study (*L. delbreuckii* sub spp. *bulgaricus NRRL B548* and *L. rhamnosus NRRL B442*) were obtained from USDA Agriculture Research Service (NRRL) Culture Collection (Peoria, IL, USA). *L. paracasei* DUP-13076 was kindly provided by A. K. Bhunia, Molecular Food Microbiology Laboratory, Food Science Department, Purdue University, West Lafayette, IN, USA. Two isolates each of *Salmonella* Enteritidis (SE; SE-21and SE-90) and multidrug resistant *S.* Typhimurium DT104 (ST; ST-43 and ST-J380), and *S.* Heidelberg (SH; SH-V6FA and SH-1) were used in the study. All bacteriological media used in the study were procured from Difco (Difco Becton, MD, USA). LAB cultures were grown in de Man, Rogosa and Sharpe broth (MRS) and *Salmonella* in Tryptic Soy broth (TSB) at 37 °C overnight. After incubation, the cultures were centrifuged (3000× *g*, 12 min, 4 °C), and washed twice in phosphate buffered saline (PBS, pH 7.0), separately. The pellet was then resuspended in PBS and used as the inoculum. Bacterial counts in the LAB and *Salmonella* cultures were confirmed following serial dilution and plating on MRS agar and tryptic soy agar (TSA), respectively. Since our preliminary experiments revealed that the LAB cultures were not adept to grow on TSA, and no significant difference (*p* > 0.05) was observed between *Salmonella* populations on TSA and xylose lysine desoxycholate agar, routine pathogen enumeration was performed using TSA.

### 3.2. Estimation of Sub-Inhibitory Concentration of LAB Cell-Free Supernatant

To obtain LAB CFSN, overnight cultures of LD/LP/LR were centrifuged (3000× *g*, 15 min, 4 °C) and the supernatant was filtered using 0.22 µm syringe filter [50]. The SIC of LAB supernatants was estimated as previously described [51,52]. Approximately 6 log_10_ CFU/mL of *Salmonella* was inoculated into 10 mL of TSB supplemented with different concentrations of CFSN (0.5%, 1%, 2.5%, 5%, 7.5%, 10%, 15%, and 20%) and incubated at 37 °C for 24 h. Following incubation, the surviving *Salmonella* population was enumerated by dilution and plating on TSA. The highest concentration of CFSN which did not inhibit *Salmonella* (SE, ST, SH) growth was determined to be the SIC.

### 3.3. Motility Assay

SE, ST, and SH isolates were cultured in Luria-Bertani broth (LB) in the presence or absence of SICs of different LAB CFSN or MRS (control) at 37 °C for 14 h. Ten microliters of the culture (8 log_10_ CFU/mL) was placed in the center of an agar plate (TSA containing 0.3% agar) and incubated for 8 h at 37 °C and zone of motility was measured [53,54]. The assay for each LAB CFSN and *Salmonella* strain was run in duplicate, and the entire experiment replicated three times.

### 3.4. Isolation of Chicken Primary CEC

Primary cecal cells were isolated according to a published protocol [35]. Briefly, ceca from healthy birds (*n* = 6) were collected in Hank’s balanced salt solution (HBSS, HyClone, Logan, UT) containing 1% PenStrep, and cecal contents were removed. Ceca were then cut into small pieces of ~3 mm^2^ and digested using a digestion media containing Dulbecco’s modified eagle medium (DMEM, HyClone), collagenase type XI, dispase I, fetal bovine serum and PenStrep (Sigma-Aldrich, St. Louis, MO, USA) for 2 h at 37 °C. The digested suspension was filtered (180 µm pore size), resuspended in 4% d-sorbitol solution, and centrifuged for 5 min at 500× *g*. The pellet was resuspended in 6% d-sorbitol, filtered (150 µm pore size) and centrifuged for 5 min at 500× *g*. The procedure was repeated until the supernatant was clear. The pellet was then resuspended in DMEM containing 2.5% heat-inactivated fetal calf serum (FCS), 0.1% insulin, 0.5% transferrin, 0.007% hydrocortisone, 0.1% fibronectin, and 1% PenStrep and incubated at 37 °C and 5% CO_2_ to form a confluent monolayer. The epithelial characteristics of the cells in the monolayer were confirmed by staining for cytokeratin (cytokeratin pan antibody (AE1/AE3), 1/2000 dilution; Thermofisher Scientific, Waltham, MA, USA) as described by Van Immerseel et al. [35]. Prior to validating the epithelial nature of the isolated CEC, cytokeratin localization in epithelial cells was confirmed by staining BATC [51]. This cell line has been previously utilized to study *Salmonella* pathogenesis in avian species [55,56,57,58].

### 3.5. Inhibition of Salmonella Adhesion to CEC

LAB cultures (LD/LP/LR) were washed and resuspended in DMEM with 10% FBS (~8 log_10_ CFU/mL). CEC were seeded on a 24-well tissue culture plate at ~10^5^ cells per well, and inoculated with ~7.0 log_10_ CFU/well of each LAB strain separately. The tissue culture trays were centrifuged at 600× *g* for 5 min, and incubated for 24 h at 37 °C in a humidified, 5% CO_2_ incubator. The cells were then washed three times with PBS and infected with ~6.0 log_10_ CFU/well of each SE, ST, or SH isolates separately (MOI 10). The tissue culture trays were centrifuged at 600× *g* for 5 min, and incubated at 37 °C in a humidified, 5% CO_2_ incubator for 2 h [59]. The wells were then washed three times with PBS, and the CEC were lysed using 0.1% Triton X-100 in PBS and incubated at 37 °C and 5% CO_2_ for 10 min to release the adherent and internalized *Salmonella*. The cell homogenates were diluted ten-fold in PBS and plated on TSA to enumerate the CEC associated *Salmonella* population [60]. In addition to *Salmonella* enumeration, LAB adherence to CEC was also estimated following dilution and plating on MRS agar. Duplicate wells were used for each treatment and control, and the experiment was repeated three times.

### 3.6. Inhibition of Salmonella Invasion in CEC

For the internalization assay, CEC monolayers were pre-exposed to the different LAB cultures and infected with SE, ST, or SH as described previously. Following infection for 2 h, monolayers were washed three times with DMEM and incubated in whole media containing 100 µg/mL gentamicin for 1 h to kill all the adhered (extra cellular) bacteria. Internalized *Salmonella* were then enumerated after triton lysis and plating on TSA [54,60]. The invasion assay for each *Salmonella* strain was run in duplicate and replicated three times.

### 3.7. Inhibition of Salmonella Invasion and Survival in HTC

Chicken monocyte cell line (HTC; [61]) was cultured in Roswell Park Memorial Institute (RPMI) 1640 Medium (HyClone) with 10% heat-inactivated fetal bovine serum and incubated at 37 °C and 5% CO_2_ to form confluent monolayers. The cells were activated and plated as described previously [12,61]. Following activation, inhibition of *Salmonella* invasion and survival in macrophages was assayed as previously described [12,54,61]. Briefly, *Salmonella* (SE/ST/SH) were grown in the presence or absence of the SICs of the LAB (LR, LP or LD) CFSN or MRS at 37 °C for 24 h. Following overnight growth, the cultures were washed and resuspended in RPMI 1640. For the internalization assay, activated and attached macrophages (10^5^ cells/well) were infected with ~6.0 log_10_ CFU/well of each SE, ST, or SH separately. The tissue culture trays were centrifuged at 600× *g* for 5 min, and incubated at 37 °C in a humidified, 5% CO_2_ incubator for 2 h. The unattached *Salmonella* were then removed by washing with RPMI, and fresh media supplemented with 100 µg/mL gentamicin was added. HTC monolayers were incubated further for an additional 1 h to kill any *Salmonella* attached to the surface. The media in the wells were changed every day with whole media containing 10 µg/mL gentamicin. The cells were washed thrice with PBS, lysed at 2, 24, 48, and 72 h using 0.1% Triton X-100 before serial dilution and plating to enumerate the surviving intracellular *Salmonella* population*.* The assay for each LAB CFSN and *Salmonella* strain was run in duplicate, and the entire experiment repeated three times.

### 3.8. Effect of LAB on Salmonella Virulence Gene Expression

SE-90, ST-43, and SH-V6FA (~6 log_10_ CFU/mL) were grown to the early stationary phase in LB broth supplemented with SICs of LAB (LR, LP or LD) CFSN or MRS at 37 °C [62]. RNA was isolated using RNeasy minikit (Qiagen, Germantown, MD, USA) according to the manufacturer’s protocol. RNA quantification was performed using the Nanodrop (Eppendorf, Enfield, CT, USA), and cDNA was synthesized using the iScript reverse transcriptase kit (Biorad, Hercules, CA, USA). cDNA was used as a template for the *Salmonella* virulence gene expression assay. Specific primers for candidate genes (*motA*, *flgG*, *hilA*, *hilD*, *sipA*, *sipB*, *invH*, *sopB* and *spvB*) were selected from the published literature [12]. The primers were custom synthesized by Integrated DNA Technologies (IDT, Coralville, IA, USA). RT-qPCR was performed on the StepOnePlus™ platform using the SYBR green assay (Applied Biosystems, Foster City, CA, USA) under custom thermal cycling conditions. Duplicate samples were used for the assay, and the experiment was repeated three times. Data were normalized to the endogenous control (16s RNA), and comparative quantification (2^−ΔΔ*C*t^) was performed to detect changes in relative gene expression between CFSN treated and untreated control (MRS) samples [63].

### 3.9. Transmission Electron Microscopy (TEM) Analysis

TEM was performed according to the published protocol with some modifications [64]. Briefly, *Salmonella* (SE-90) grown in TSB supplemented with or without SIC of LR CFSN was centrifuged for 3000× *g* for 15 min, and the pellet was suspended in a fixative solution composed of 1.5% glutaraldehyde, 1.5% paraformaldehyde, 0.12 M phosphate buffer, and 3 mM magnesium chloride. Two percent molten agarose was poured onto the pellet and allowed to harden. The embedded pellet was then washed thrice with phosphate buffer (0.1 M phosphate buffer and 3 mM magnesium chloride) for 15 min each by slow rolling. The bacteria were fixed in 2% osmium tetroxide in 0.1 M phosphate buffer and 3 mM magnesium chloride for an hour. Dehydration and clearing were performed for 10 min each in distilled water, 30%, 50%, 70%, 95%, and 100% ethanol, and finally in acetone. Embedment in the resin was done using one part resin and one part acetone for 2 h, two part epon/araldite resin and one part acetone for 17 h, and 100% resin for 3, 2.5, and 1.5 h. Finally, the pellet was infiltrated with pure resin, incubated at 60 °C for 48 h. For imaging, ultrathin sections (100 nm) of the resin block was transferred to 200 mesh copper grids and stained using 3% Sato’s lead citrate and 0.5% uranyl acetate. TEM images were taken using an FEI Tecnai™ microscope (FEI, Hillsboro, OR, USA).

### 3.10. Statistical Analysis

Log_10_ values of *Salmonella* count at different time periods were tested for significance at a *p* value of <0.05 using the PROC GLIMMIX procedure of SAS (version 9.2; SAS Institute Inc., Cary, NC, USA). For motility, adhesion, invasion, survival in HTC and RT-qPCR assays, the data were analyzed using the PROC-MIXED procedure of SAS. The results of two independent tests were tested for significance at a *p* value of <0.05 using MANOVA.

## 4. Conclusions

In summary, this study demonstrated the anti-*Salmonella* properties of select LAB cultures (LR, LP, and LD). The LAB isolates were able to adhere to intestinal epithelial cells and competitively exclude the pathogen. More specifically, the reduction in pathogen colonization (in vitro) was mediated by a significant inhibition of *Salmonella enterica* (*S.* Enteritidis 21 and 90, *S.* Typhimurium DT 104 43 and J380, and *S.* Heidelberg V6FA and 1) motility, attachment and invasion in CEC, and survival in chicken macrophages. In addition, our results revealed that LR, LP, and LD exerted their effects via the modulation of virulence gene expression in *Salmonella*. Furthermore, they were also found to be effective against multi-drug-resistant *S. enterica* serovars, namely *S.* Typhimurium DT104 and *S.* Heidelberg. Therefore, *L. delbreuckii* sub sp. *bulgaricus* NRRL B548, *L. rhamnosus* NRRL B442, and *L. paracasei* DUP-13076 could be potentially used to control *Salmonella* in chicken. However, further in vivo validation of these probiotic isolates in live birds is to be conducted.

## Figures and Tables

**Table 1:** Effect of sub-inhibitory concentrations of LAB supernatants on the expression of virulence genes in *Salmonella* Enteritidis, *S*. Typhimurium, and *S*. Heidelberg.

Treatments	*invH*	*hilA*	*hilD*	*sipA*	*sipB*	*sopB*	*spvB*
SE Ctrl	1 ^a^	1 ^a^	1 ^a^	1 ^a^	1 ^a^	1 ^a^	1 ^a^
SE + LR	–1.85 ± 0.08 ^b^	–4.58 ± 1.13 ^b–d^	–2.90 ± 0.15 ^b,c^	–5.01 ± 0.76 ^b–d^	–18.69 ± 0.46 ^g^	–6.67 ± 1.39 ^b–d^	–8.17± 1.55 ^b–d^
SE + LD	–2.66± 0.14 ^b,c^	–3.54 ± 0.71 ^b,c^	–2.20 ± 0.10 ^b^	–1.87 ± 0.15 ^b^	–1.91 ± 0.19 ^b^	–2.93 ± 0.44 ^b,c^	–17.27 ± 3.26 ^f,g^
SE + LP	–4.07± 0.84 ^a,b^	–4.07 ± 0.38 ^b–d^	–2.81 ± 0.28 ^b,c^	–11.19± 0.36 ^d,e^	–27.19 ± 0.66 ^i^	–15.70± 0.54 ^e–g^	–25.46 ± 0.91 ^h,i^
ST Ctrl	1 ^a^	1 ^a^	1 ^a^	1 ^a^	1 ^a^	1 ^a^	1 ^a^
ST + LR	–11.31± 0.22 ^d,e^	–2.31 ± 1.04 ^b,c^	–2.74 ± 0.9 ^b,c^	–2.89 ± 1.12 ^b^	–19.35 ± 0.27 ^g^	–3.55 ± 0.51 ^b,c^	–7.16 ± 0.88 ^b–d^
ST + LD	–3.98 ± 1.2 ^a,b^	–3.98 ± 0.93 ^b–d^	–2.09 ± 0.66 ^b^	–1.37 ± 0.28 ^b^	–1.78 ± 0.28 ^b^	–1.89 ± 0.28 ^b^	–10.40 ± 1.17 ^d,e^
ST + LP	–3.05± 0.77 ^b,c^	–1.45 ± 0.56 ^b^	–2.72 ± 0.55 ^b,c^	–12.13± 1.65 ^d–f^	–28.92 ± 1.53 ^i^	–6.00 ± 1.93 ^b–d^	–25.46 ± 4.8 ^h,i^
SH Ctrl	1 ^a^	1 ^a^	1 ^a^	1 ^a^	1 ^a^	1 ^a^	1 ^a^
SH + LR	–3.12 ± 0.64 ^b,c^	–2.00 ± 0.2 ^a^	–2.54 ± 0.6 ^b,c^	–3.29 ± 0.16 ^b,c^	–17.21 ± 3.45 ^f,g^	–3.22 ± 0.33 ^b,c^	–19.97 ± 0.22 ^g,h^
SH+ LD	–4.42 ± 1.09 ^a,b^	–5.76 ± 0.55 ^b–d^	–2.04 ± 0.63 ^b^	–1.67 ± 0.17 ^b^	–3.29 ± 0.75 ^b,c^	–2.22 ± 0.23 ^b^	–19.61 ± 0.53 ^g,h^
SH + LP	–5.45 ± 2.53 ^a,b^	–12.11± 0.55 ^d–f^	–2.68 ± 0.22 ^b,c^	–10.53 ± 0.99 ^d,e^	–27.96 ± 1.99 ^i^	–4.69 ± 0.67 ^b–d^	–30.86 ± 1.41 ^i^

^a−i^ Different superscripts indicate the significant difference in LS-means (*p* < 0.05).
